# Saturation Behavior: a general relationship described by a simple second-order differential equation

**DOI:** 10.1186/1742-4682-7-11

**Published:** 2010-04-13

**Authors:** Gordon R Kepner

**Affiliations:** 1 Membrane Studies Project, PO Box 14180, Minneapolis, MN 55414, USA

## Abstract

**Background:**

The numerous natural phenomena that exhibit saturation behavior, *e.g*., ligand binding and enzyme kinetics, have been approached, to date, via empirical and particular analyses. This paper presents a mechanism-free, and assumption-free, second-order differential equation, designed only to describe a typical relationship between the variables governing these phenomena. It develops a mathematical model for this relation, based solely on the analysis of the typical experimental data plot and its saturation characteristics. Its utility complements the traditional empirical approaches.

**Results:**

For the general saturation curve, described in terms of its independent (*x*) and dependent (*y*) variables, a second-order differential equation is obtained that applies to any saturation phenomena. It shows that the driving factor for the basic saturation behavior is the probability of the interactive site being free, which is described quantitatively. Solving the equation relates the variables in terms of the two empirical constants common to all these phenomena, the initial slope of the data plot and the limiting value at saturation. A first-order differential equation for the slope emerged that led to the concept of the effective binding rate at the active site and its dependence on the calculable probability the interactive site is free. These results are illustrated using specific cases, including ligand binding and enzyme kinetics. This leads to a revised understanding of how to interpret the empirical constants, in terms of the variables pertinent to the phenomenon under study.

**Conclusions:**

The second-order differential equation revealed the basic underlying relations that describe these saturation phenomena, and the basic mathematical properties of the standard experimental data plot. It was shown how to integrate this differential equation, and define the common basic properties of these phenomena. The results regarding the importance of the slope and the new perspectives on the empirical constants governing the behavior of these phenomena led to an alternative perspective on saturation behavior kinetics. Their essential commonality was revealed by this analysis, based on the second-order differential equation.

## Background

This paper answers the question: is there a general mathematical model common to the numerous natural phenomena that display identical saturation behavior? Examples include ligand binding, enzyme kinetics, facilitated diffusion, predator-prey behavior, bacterial culture growth rate, infection transmission, surface adsorption, and many more. The mathematical model developed here is based on a general second-order differential equation (D.E.), free of empirical constants, that describes the basic relation underlying these saturation phenomena [1].

A common and productive way to analyze a specific saturation phenomenon uses a model for the proposed mechanism. This leads to an algebraic relation that describes the experimental observations, and helps interpret features of the mechanism. Where the phenomenon involves chemical reactions, for example, the models rely on assumptions about reaction mechanisms, dissociation constants, and mass action rate constants [2-7]. Note that such mechanisms cannot be proved definitively by standard kinetic studies [8].

In view of the ubiquity of saturation phenomena, it seems useful to seek one mathematical model that describes all such phenomena. The model presented here relies solely on the basic mathematical properties of the experimentally observed data plot for these phenomena--the independent variable versus the dependent variable. It is free of mechanism and therefore applies uniformly to all these phenomena. The analysis starts with a second-order differential equation, free of constants, that offers a general way of describing them. This equation is then integrated and applied to illustrative examples.

## Results

### Basic saturation behavior case

The general nature of the initial extensive mathematical analysis suggests using familiar mathematical symbols-- *x*, *y*, *dy*, *dx*, *dy*/*dx*, *d*^2^*y*/*dx*^2^, etc.--instead of using the symbols and notation particular to a specific saturation phenomenon, such as ligand binding where *x *would be *A *(free ligand), and *y *would be *A*_b _(bound ligand). One can then substitute any phenomenon's particular symbols into the key equations.

**Figure 1:**
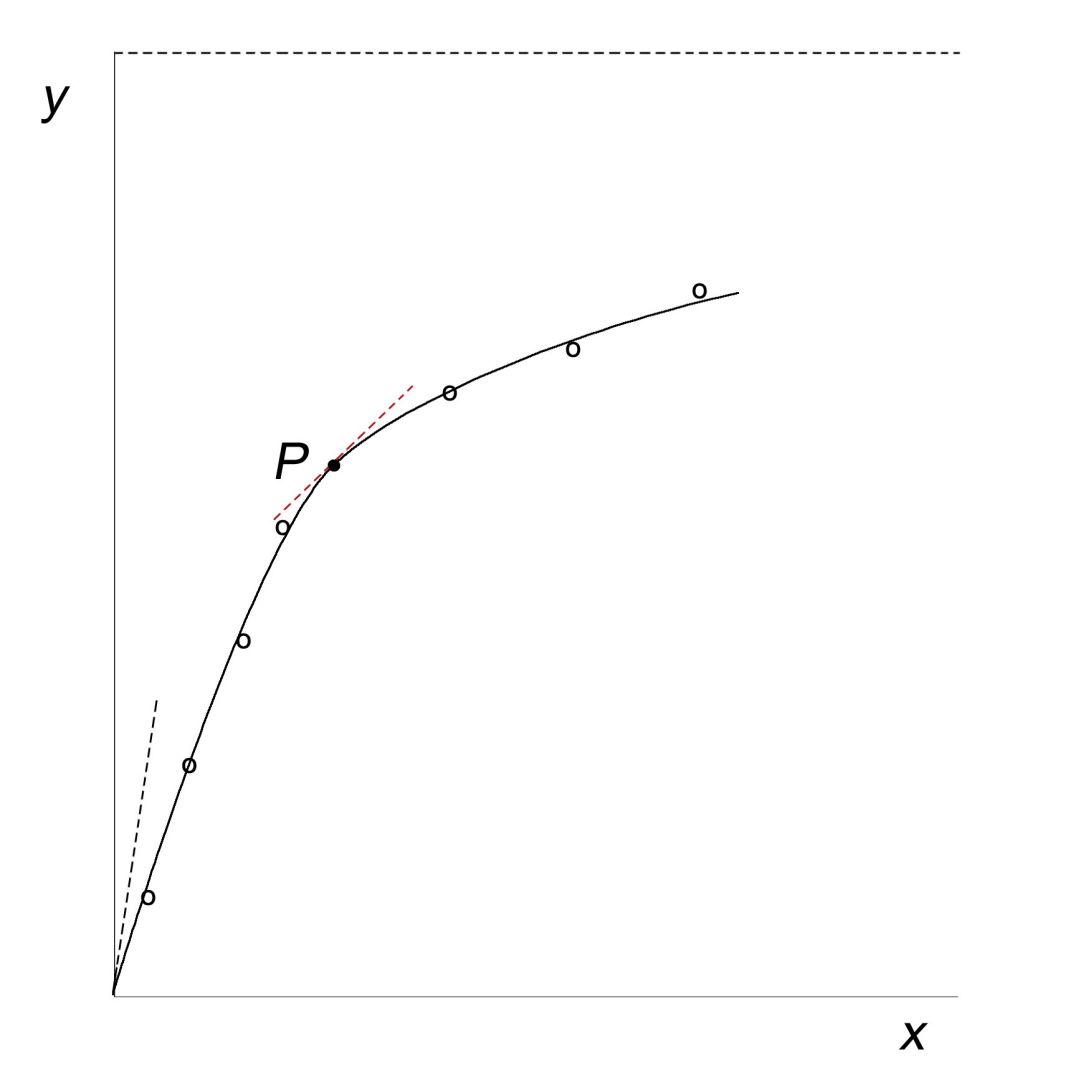
**Typical idealized experimental data plot for those natural phenomena showing saturation behavior**. The black dashed line is the initial slope, (*dy*/*dx*)_0_, and the red dashed line is the tangent at point *P*, (*dy*/*dx*)_P_.

A typical experimental data plot for these natural phenomena that exhibit saturation behavior is shown in Figure 1. Its *essential feature *is that each successive incremental increase, *dx*, in *x *is less effective at increasing *dy*. At very large values of *x *(saturation), the plot approaches its limiting value, the asymptote. As *x *increases: the fractional changes (*dx*/*x *and *dy*/*y*) decrease; the slope (*dy*/*dx*) is positive, steadily decreasing, and continuous; the second derivative (*d*^2^*y*/*dx*^2^) is steadily decreasing, and negative--because the tangent at *P *is above the curve. Thus, (*d*^2^*y*/*dx*^2^) = -|*d*^2^*y*/*dx*^2^|.

The following generalized D.E. leads to many different mathematical relations, depending on the particular integer values of N and M. These describe, collectively, numerous natural phenomena.(1)

Note that each term takes the fractional change form. It will be shown here, for N = M = 2, that this yields the second-order D.E., free of empirical constants, that gives the mathematical relation *y *= *a*·*x*/(*b *+*x*). This relation describes the saturation plot of Figure 1. Integration and analysis then lead to the definitions of the basic empirical constants that describe all saturation plots. Setting *κ *= *dy*/*dx *= slope gives(2)

where *dκ*/*κ *is the fractional change in the slope.

Integrating and taking anti-logarithms gives the first-order D.E. for the slope,(3)

Integrating again and rearranging gives(4)

This algebraic relation, when substituted into equation (1), satisfies the second-order D.E. Therefore, it is a general solution. The system constants are determined by forcing the general solution to fit the physical boundary conditions (*x *→ 0 and *x *→ ∞), giving a unique solution.

Evaluate *C*_1 _and *C*_2 _using equation (4). Let *x *→ 0, so that *C*_1 _>>*C*_2_·*x*, and therefore(5)

Rearranging so *y *= 1/[(*C*_1_/*x*) + *C*_2_], let *x *→ ∞, then *C*_2 _= 1/*y*_∞ _= 1/*Υ*_sat_, where *Υ*_sat _is the limiting value as *y *approaches the asymptote (saturation). Thus(6)

This equation defines the roles of the two directly measurable and independent empirical constants of the experimental system, *κ*_0 _and *Y*_sat_. Rearranging equation (6) gives(7)

the general form of the standard algebraic relation used to describe the data plot in Figure 1, [2-7,9-12].

These saturation phenomena are typified by the binding of a substance (e.g., a ligand or substrate) to a binding site. This can be analyzed in terms of random interactions between *x *and the binding site. In general, *y*_x_/*Υ*_sat _= Γ_bd_, the fraction bound, which can be equated to the probability the site is occupied, for a given value of *x*. Thus Γ_bd _= *x*/(*K *+ *x*) = *κ*_0_·*x*/[*Y*_sat _+ (*κ*_0_·*x*)]. The probability the site is free is Γ_fr _= 1 - Γ_bd_, so(8)

Thus, as *x *→ 0, Γ_fr _→ 1, and as *x *→ ∞, Γ_fr _→ 0.

Define Δ to mean *the change in*. Then the (change in slope)/(slope) equals Δ (*dy*/*dx*)/(*dy*/*dx*). Let Δ (*dy*/*dx*) = (1/2)·(*d*^2^*y*/*dx*^2^)·*dx*. The average slope is (*y*/*x*). Thus, Δ (*y*/*x*) = *d *(*y*/*x*), where *d *(*y*/*x*)/(*y*/*x*) = (*dy*/*y*) - (*dx*/*x*). Rearranging equation (1) with N = M = 2, and substituting equations (7) and (8) into it, yields(9)

Thus, the change in the slope (*dy*/*dx*) divided by the change in the average slope (*y*/*x*) is determined by Γ_fr_.

Substituting into equation (3) for the slope gives(10)

### Ligand binding

Consider a small molecule, the ligand, that is present in either the free form, *A*, or the bound form, *A*_b_. For the simplest case, assume that each ligand binds to a single specific binding site (bs). This could be on a macromolecule, *M*_bs_, such as a protein. These sites are presumed to be independent and to have the same binding constant. The details of the experimental conditions required for these binding studies are found in standard reference texts [2,5,7,9-12].

The basic overall binding reaction is defined to be

The necessary and sufficient condition for this analysis is the experimental data plot of *A*_b _versus *A*. In Figure 1, set *y *= *A*_b _and *x *= *A*. Then substitute into the key equations, for example, equation (6).(11)

The total number of binding sites in the experimental system (*M*_bs_, *A*, *A*_b_) is (*A*_b_)_sat_. It is the limiting amount of ligand binding observed at saturation with *A*. The initial slope is *κ*_0_, the system's limiting *binding rate *when *A *→ 0, and Γ_fr _→ 1. Thus, (*A*_b_)_sat _and *κ*_0 _are the empirical constants of the ligand binding system. The conventional models of the binding mechanism identify *K*_d _as the dissociation constant in mol L^-1 ^[2,5,6,10-12]. Equation (11) is often referred to as the Langmuir adsorption isotherm, or the Hill binding equation. It is sometimes written using the binding fraction, Γ_bd _= *A*_b_/(*A*_b_)_sat_.

The units of *κ*_0 _= *k*_bind_·(*A*_b_)_sat_, where *k*_bind _= 1/*K*_d_, are

Thus, *k*_bind _is the binding rate constant for one mole of binding sites evaluated at *A *→ 0, where Γ_fr _→ 1. It characterizes the binding strength of the ligand for the binding site. Therefore, a high value of *k*_bind _means a high value of the initial slope of the system, *κ*_0_, and so the value of *K*_d _is decreased.

The complete expression for the units of *κ*_0 _illustrates how descriptive information could be lost when units are cancelled. Thus, the units [mol L^-1 ^of (*dA*_b_) bound/mol L^-1 ^of (*dA*) added]_0 _describe a useful aspect of the binding process--the fraction of the added (*dA*) that is bound [(*dA*_b_)/*dA*], as *A *→ 0. Taken over one minute, this yields the binding rate constant for one mole of binding sites.

The slope is given by(12)

where Γ_fr _= (*A*_b_)_sat_/[(*A*_b_)_sat _+ (*κ*_0_·*A*)]. Thus, *κ*_A _is defined as the system's effective binding rate at any value of *A*--to distinguish it from the highest value of *κ*, when *A *→ 0, giving *κ*_0_, the system's limiting binding rate. Thus, if *κ*_0 _is increased, then the ligand binding increases and (Γ_fr_)_A _decreases, for a given value of *A*, because now more of the sites are occupied at *A*. If (*A*_b_)_sat _is doubled, for example, Γ_fr _will be increased--but not proportionately, see equation (11).

**Figure 2:**
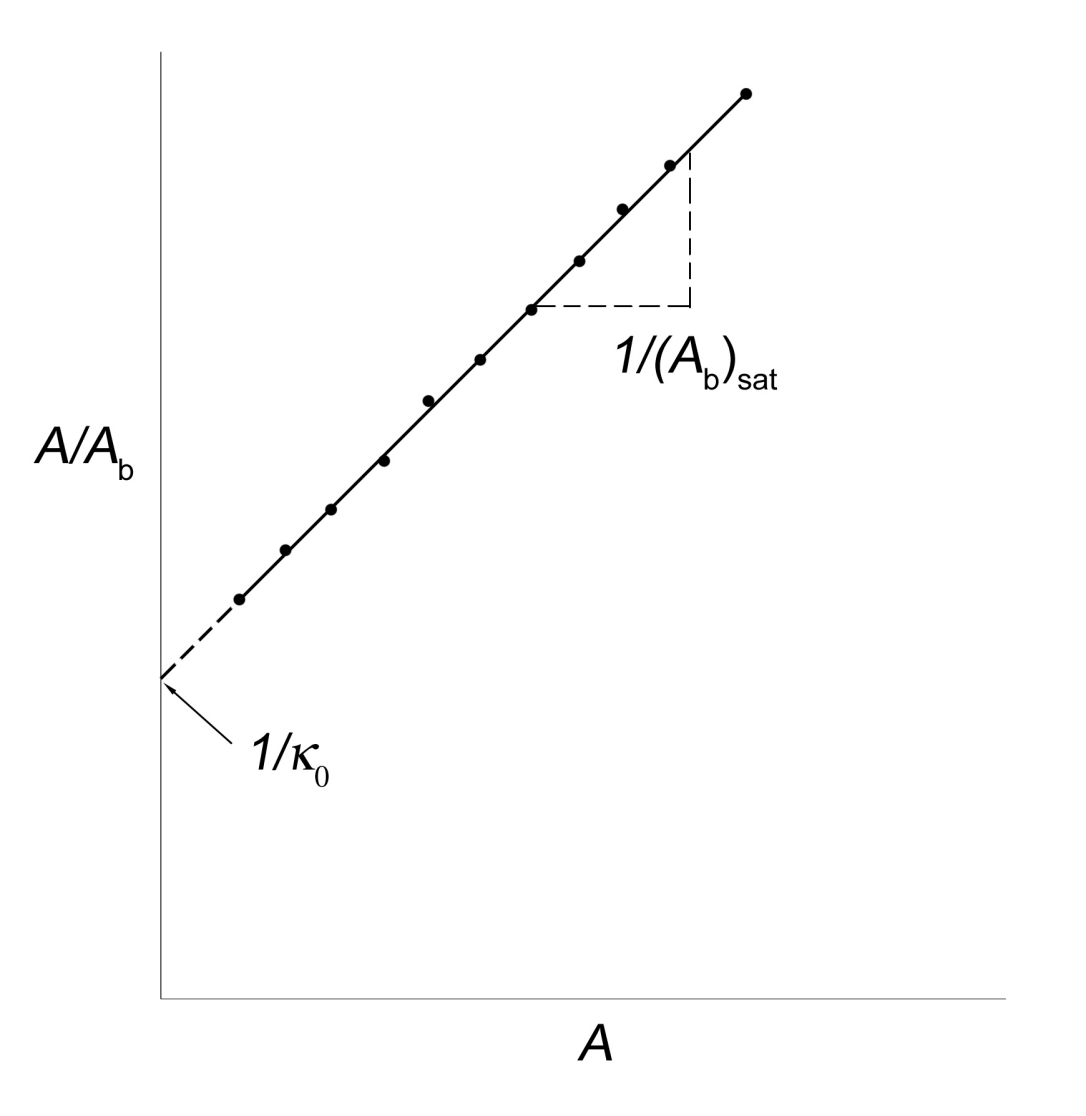
**Typical plot of idealized experimental data to facilitate calculation of the empirical constants, (*A*_b_)_sat _and *κ*_0_**.

Knowing the value of *κ*_0_, obtained from experimental data, one can calculate Γ_fr _for any *A*. Also, , using equation (12), which shows how the slope depends on (1/*A*^2^). The values of *κ*_0 _and (*A*_b_)_sat _can be obtained directly by using a standard linear transformation of the data plot, see the enzyme kinetics case, equation (19). This gives a plot of (*A*/*A*_b_) versus *A*, with a slope of 1/(*A*_b_)_sat_, and ordinate intercept of 1/*κ*_0_, see Figure 2.

### Other examples

The term, binding site, is used for convenience as a general way of identifying the interactive locus of many saturation phenomena. For example: ligand binds to a macromolecule; a nutrient molecule binds to a receptor on a bacterial membrane and is transported inside; a prey is bound to a predator's jaws; a substrate binds to an enzyme's catalytic site; a molecule is adsorbed at sites on a surface (Langmuir's adsorption). Some saturation phenomena are less well-suited to this binding site characterization--e.g., the stock-recruitment model for producing new fish biomass from spawning stock [13].

The simplest case of bacterial growth in a chemostat shows saturation dependence on the available nutrient [14,15]. Thus, *dr*/*dA *= *κ*_A _= (1/*κ*_0_)·(*r*/*A*)^2 ^= , so(13a)

where *r *is the experimentally measured bacterial growth rate (*g*·L^-1 ^min^-1^), at a given concentration of nutrient, *A*(*g*·L^-1^). *R*_sat _is the limiting growth rate at saturation with nutrient (*g*·L^-1 ^min^-1^). So, *K *= *R*_sat_/*κ*_0 _in *g*·L^-1^, where the initial slope is *κ*_0 _(grams bacteria·L^-1 ^min^-1^/grams nutrient·L^-1^), evaluated at *A *→ 0. It measures the effectiveness of the specific bacteria's ability to convert a specific nutrient to bacterial growth--when all the receptor sites on the bacterial membrane are available. Thus, different bacteria using the same nutrient would have different values of *κ*_0_, reflecting the relative effectiveness of nutrient binding to the different receptor sites.

Consider predator-prey behavior in the simple case of the functional response model, where the attack rate increases, but at a decreasing rate with increased prey density [15-18]. Here, *dn*/*dA *= *κ*_A _= (1/*κ*_0_)·(*n*/*A*)^2 ^= , so(13b)

where *n *is the number of prey attacked over unit time by the predators present, and *N*_sat _is the limiting rate of attack at saturation with prey. Set *A *equal to the prey density (e.g., number of prey per square kilometer). Then *K *= *N*_sat_/*κ*_0_, where the initial slope, *κ*_0_, measures the effectiveness of the predator attacking the prey, as *A *→ 0. Thus, a predator attacking two different prey yields different values of *κ*_0_.

### Michaelis-Menten (M-M) enzyme kinetics

The basic overall enzymatic reaction is the conversion of one substrate molecule, *A*, to one product molecule, *P*, by an enzyme molecule, *E*, that catalyzes this conversion at its catalytic site (cs).

The necessary and sufficient condition for this analysis is the experimental data plot of (*dP/dt*) = *p*, versus *A*. See Figure 1, where *p *= *y *and *A *= *x*. The experimental conditions required for measuring *p *and *A *are described in standard reference texts [2-4,7,9-12]. The use here of *p*, instead of the conventional *v*, focuses attention on the actual measured quantity and how it relates to *A*, in terms of *dp*/*dA *and *d*^2^*p*/*dA*^2^.

Equation (9) becomes Δ (*dp*/*dA*)/Δ (*p*/*A*) = Γ_fr_, and equation (1) gives(14)

Thus, the slope at any point, *A*, is *κ*_A _= *κ*_0_·(Γ_fr_)^2^, where, from equation (8), Γ_fr _= *P*_sat_/[*P*_sat _+ (*κ*_0_·*A*)]. Note that *κ*_A _equals [(*dA*)converted/(*dA*)added]_A _= (*dp*/*dA*)_A_. This could be viewed as a measure of how effectively the system is converting substrate to product at *A*. It decreases rapidly as (1/*A*^2^).

Equation (6) becomes(15)

The initial slope is *κ*_0_, and *P*_sat _is the limiting rate of enzyme catalysis when saturated with *A*. Increasing *κ*_0 _will increase the binding of *A *to the catalytic site. These are the two independent empirical constants of the experimental system (*E*_t_, *A*, *P*). Equating (*P*_sat_/*κ*_0_) to *K*_m_, the Michaelis constant, and with *p *= *v*, and *P*_sat _= *V*, gives(16)

the standard form for the M-M equation of enzyme kinetics.

Note that *P*_sat _≡ *k*_cat_·*E*_t_, where *E*_t _is the total enzyme concentration present experimentally, which may not be known. The catalytic constant, *k*_cat_, is the limiting catalytic rate at which one mole of enzyme molecule could operate if completely saturated with substrate. Similarly, *κ*_0 _≡ *k*_bind_·*E*_t_, where *k*_bind _is here defined to be the *binding rate constant *of the substrate for the catalytic sites on one mole of enzyme (min^-1 ^mol^-1^) -- evaluated when *A *→ 0, where the catalytic site is maximally available, because Γ_fr _→ 1.

Equation (8) can be rewritten to give(17)

Thus, if *k*_bind _is increased, Γ_fr _is decreased, because there are fewer free sites available at a given value of *A*. Whereas, if *k*_cat _is increased, Γ_fr _is increased. The increased turnover rate means more free sites are available at a given value of *A*. As expected, Γ_fr _is independent of *E*_t_, because Γ_fr _depends only on the basic properties of the enzyme's catalytic function, *k*_bind _and *k*_cat_.

Enzyme kinetics differs from ligand binding because there is also a conversion step. The binding step is much faster than the conversion step, where the catalytic site converts the bound substrate to product and releases it. This is commonly assumed to involve a simple 1:1 stoichiometric relation between substrate bound and product released [19]. The binding rate constant for one mole of enzyme is defined here to be = *κ*_0_/*E*_t _= *k*_cat_/*K*_m_. The ratio, *k*_cat_/*K*_m_, is often referred to as the specificity constant [19,20]. Thus, *k*_bind _indicates the strength of the mutual interaction between a specific substrate and a specific enzyme, at the catalytic site, measured when *A *→ 0. It defines a collective property for each particular combination of substrate and enzyme. For example, let *A *and *E*_cs _→ *k*_bind_, while *A*' and *E*'_cs _→ *k*'_bind_, where *k*_bind _most probably differs from *k*'_bind_, but might not. Therefore, the higher the value of *k*_bind_, the more effectively does the substrate bind to the enzyme's catalytic site. The enzyme and substrate, taken together, perform better at higher values of *k*_bind _[20].

Thus, *k*_bind _and *k*_cat _can be considered the basic properties of this single enzyme molecule's catalytic function. So(18)

Therefore, *K*_m _is defined by the ratio of the experimental system's empirical constants, which depend on the enzyme's basic properties. When *E*_t _is known, one can obtain values for *k*_cat _and *k*_bind_. Whereas, although *P*_sat _and *κ*_0 _often are measured experimentally where *E*_t _is not known, their ratio still gives *K*_m_. Doubling *E*_t _will double both *P*_sat _and *κ*_0_, so the ratio, *K*_m_, remains unchanged.

For clarity and convenience, the definitions and units of the various constants are explicitly stated here.

There are various standard linear transformations of equations (15) and (16) that aid in the initial analysis of the data plot in Figure 1, [4-6]. Equation (19) is one.(19)

This gives a linear plot of (*A*/*p*) versus *A*. The slope is (1/*P*_sat_) and the ordinate intercept is (1/*κ*_0_), recall Figure 2. This provides direct evaluation of the system's empirical constants, *κ*_0_, and *P*_sat_, from the experimental data. Using *C*_1 _= 1/*κ*_0_, obtained from equation (19), one can calculate *κ*_A_, at any value of *A*, using the equation for *κ*_A_.

## Discussion

### Basic case

The mathematical model presented here is based solely on the observed experimental data plot for these phenomena, as shown in Figure 1. This analysis of the second-order D.E. offers an alternative approach, free of mechanism, that describes the common process underlying all natural phenomena exhibiting saturation behavior. It provides a general mathematical description of these phenomena. The D.E. approach takes a path of discovery that reveals the salient features of these phenomena on the way to reaching *y *= *x*/[(1/*κ*_0_) + (1/*Y*_sat_)·*x*]. It complements approaches that model each specific saturation phenomenon separately, in terms of a proposed mechanism.

The D.E. analysis provided two general integration constants, *C*_1 _and *C*_2_, evaluated at the known boundary conditions, *x *→ 0 and *x *→ ∞. This gave the two empirical constants, *κ*_0 _and *Y*_sat_, that defined the relation between the variables of any saturation phenomenon -- see equation (6), the general algebraic description of these saturation phenomena. The empirical constant, *κ*_0_, the initial slope, and its practical significance, have not been recognized previously.

Applying the quantitative relation for Γ_fr _clarified the functioning of the interactive site. It showed that the underlying relation describing these phenomena, equation (1), became Δ(*dy*/*dx*)/Δ(*y*/*x*) = Γ_fr_, see equation (9). The slope, equation (3), became *κ *= *κ*_0_·(Γ_fr_)^2^. Its strong dependence on (1/*x*^2^) was shown. As *x *increases, each added increment, *dx*, sees a lower Γ_fr_, because a greater fraction of the sites are occupied at the instant of adding *dx*. This leaves fewer sites free to attend to the conversion of this additional *dx*. This behavior is the essence of how these saturation phenomena function in response to increased *x*.

### Ligand binding, bacterial growth, predator-prey

The response of these saturation phenomena to increased *A *is driven by Γ_fr_, see equations (9) and (10). The independent empirical constants for each phenomenon relate the variables of each and define the *K *that characterizes each one, see equations (11), (13a) and (13b). This mathematical model defines *K*, in general, as the ratio of the limiting rate/initial slope. Figure 2 shows how to obtain their values from the data. Other applications of this general approach include surface adsorption, facilitated transport, and transmission of infection. It emphasizes the utility of the initial slope, *κ*_0_.

### Michaelis-Menten enzyme kinetics

Equation (10) shows that the slope, *κ*_A_, depends on (Γ_fr_)^2 ^and (1/*A*^2^). Thus, Γ_fr _drives the experimental system's behavior and accounts, quantitatively, for the decrease in the slope with increasing *A*. This leads to the concepts of:

■ the system's effective binding rate, for *E*_t _moles of enzyme, at *A*.

■ the binding rate constant for one mole of enzyme, at *A *→ 0.

For *E*_t _moles, (*dp*/*dA*)_0 _= *κ*_0_, and for one mole, (*dp*/*dA*)_0_/*E*_t _= *κ*_0_/*E*_t _= *k*_bind_. Note that *κ*_A _= (*dp*/*dA*)_A _can be calculated using the equation for the slope and equation (19). Thus, the (slope)_A_/(slope)_0 _= (Γ_fr_)^2^, where Γ_fr _= *P*_sat_/[*P*_sat _+ (*κ*_0_·*A*)].

The D.E. analysis defined the two independent empirical constants of this experimental system as *κ*_0 _and *P*_sat_. Equation (15) is the general algebraic relation for illustrating their independent roles. Equation (18) ties together these empirical constants and the basic properties, *k*_cat _and *k*_bind_, to relate them to *K*_m_. Thus, *k*_bind _and *k*_cat_, taken together, can expand the ability to characterize and compare the interaction of enzymes and their substrates.

The usual model for the M-M enzyme reaction mechanism defines *K*_m _as a constant derived from the reaction rate constants. Such models are essential in pursuing the details of a proposed mechanism for M-M enzyme reactions, or for any saturation phenomenon. Yet, numerous different interpretations of what *K*_m _means have arisen in the literature, based on the standard model and mechanism. Some examples include: parameter, kinetic constant, not an independent kinetic constant, empirical quantity, a constant for the steady-state, measures affinity in the steady-state, should not be used as a measure of substrate affinity, most useful fundamental constant of enzyme chemistry, not a true equilibrium constant, dubious assertion that *K*_m _reflects an enzyme's affinity for its substrate [2-12,21]. According to Riggs, "Notice that the Michaelis constant is not a rate constant, nor an affinity constant, nor a dissociation constant, but is merely a constant of convenience" [22]. The interpretation presented here, based on the mathematical model, is rooted in equation (18). It showed that *K*_m _= *k*_cat_/*k*_bind_, the ratio of the enzyme's basic properties. Thus, this model viewed *K*_m _as a derived quantity, and not as an independent basic property of the enzyme molecule's catalytic function.

The action of enzyme inhibitors offers additional perspective on interpreting *K*_m _= *k*_cat_/*k*_bind_. Consider five basic cases of enzyme inhibition: Competitive, Uncompetitive, Pure Non-Competitive, Predominantly Competitive, Predominantly Uncompetitive [19]. In no case does the inhibitor (*I*) cause the limiting rate, *P*_sat_, or the initial slope, *κ*_0_, of the observed data plot for the experimental system (*E*_t_, *I*, *A*, *P*) to *increase*. The value of *K*_m _= *k*_cat_/*k*_bind_, however, is observed to *increase*, remain unchanged, or decrease--depending on the *relative *effects of the inhibitor on *k*_cat _and *k*_bind_. Any basic property of an enzyme molecule's catalytic function should never *increase *in the presence of an inhibitor. Thus, *k*_cat _and *k*_bind _meet this condition. Their ratio, *k*_cat_/*k*_bind _= *K*_m_, does not. Therefore, *K*_m _is not one of the two basic properties. Changing from *K*_m _to *A*_M_, the Michaelis concentration, would be consistent with this interpretation [19].

The ratio of these observable empirical constants, *P*_sat_/*κ*_0_, defines *K*_m_. Thus, the mathematical analysis offers an operational definition of *K*_m_, independent of any interpretations [19]. This approach to defining *K*_m _is consistent with all the known factors. "Definitions based on what is actually observed are therefore on a sounder and more lasting basis than those that depend on an assumed mechanism" [19]. Numerous mechanisms can generate M-M kinetics; "Consequently there is no general definition of any of the kinetic parameters... in terms of the rate constants for the elementary steps of a reaction's mechanism" [19].

The algebraic relation, *p *= *P*_sat_·*A*/(*K*_m _+ *A*), describes the data plot of an enzyme kinetic study. Its validity is independent of any mechanism. The mechanism-free approach derives this algebraic relation directly from the second-order D.E. This general analysis also reveals the underlying factors, such as Γ_fr_, that govern the basic behavior of these saturation phenomena.

The enzyme's catalytic function involves two distinct processes, binding the substrate and converting it to product. This mathematical analysis demonstrated that the two empirical constants of the D.E., *κ*_0 _= *E*_t_·*k*_bind _and *P*_sat _= *E*_t_·*k*_cat_, define these two processes--binding and catalysis--in terms of the basic properties, *k*_bind _and *k*_cat_.

## Conclusions

The results presented here are completely general and based entirely on a mathematical model that analyzes the observed experimental data plot for the relation between the independent and dependent variables. They apply directly to every natural phenomenon displaying the characteristic saturation behavior that produces the hyperbolic kinetics described by the relation, . The second-order D.E. presented here reveals the basic underlying relation that applies to these phenomena and its dependence on the probability a site is free. The analysis provides a theoretical basis for defining the empirical constants and basic properties of each saturation phenomenon, based on the two constants of integration evaluated at the boundary conditions. The universality of these saturation phenomena makes it useful to have the D.E., free of constants, that describes the basic properties of all these systems.

The first-order D.E. derived here introduces the concept of the effective binding rate. It is directly related to the slope of the experimental data plot. The initial slope is where it is highest, the binding rate constant of the ligand for the binding site. The analysis revealed the significance of the initial slope as an independent empirical constant for these systems exhibiting saturation behavior, and its role in determining the probability that the active site is free.

## Competing interests

The author declares that he has no competing interests.
